# A review of geospatial methods for population estimation and their use in constructing reproductive, maternal, newborn, child and adolescent health service indicators

**DOI:** 10.1186/s12913-021-06370-y

**Published:** 2021-09-13

**Authors:** Kristine Nilsen, Natalia Tejedor-Garavito, Douglas R. Leasure, C. Edson Utazi, Corrine W. Ruktanonchai, Adelle S. Wigley, Claire A. Dooley, Zoe Matthews, Andrew J. Tatem

**Affiliations:** 1 WorldPop, School of Geography and Environmental Science, University of Southampton, Southampton, UK; 2 Department of Social Statistics and Demography, University of Southampton, Southampton, UK

**Keywords:** RMNCAH, Subnational estimation, Universal coverage, Denominators, Gridded data sets, Geospatial modelling

## Abstract

**Background:**

Household survey data are frequently used to measure reproductive, maternal, newborn, child and adolescent health (RMNCAH) service utilisation in low and middle income countries. However, these surveys are typically only undertaken every 5 years and tend to be representative of larger geographical administrative units. Investments in district health management information systems (DHMIS) have increased the capability of countries to collect continuous information on the provision of RMNCAH services at health facilities. However, reliable and recent data on population distributions and demographics at subnational levels necessary to construct RMNCAH coverage indicators are often missing. One solution is to use spatially disaggregated gridded datasets containing modelled estimates of population counts. Here, we provide an overview of various approaches to the production of gridded demographic datasets and outline their potential and their limitations. Further, we show how gridded population estimates can be used as alternative denominators to produce RMNCAH coverage metrics in combination with data from DHMIS, using childhood vaccination as examples.

**Methods:**

We constructed indicators on the percentage of children one year old for diphtheria, pertussis and tetanus vaccine dose 3 (DTP3) and measles vaccine dose (MCV1) in Zambia and Nigeria at district levels. For the numerators, information on vaccines doses was obtained from each country’s respective DHMIS. For the denominators, the number of children was obtained from 3 different sources including national population projections and aggregated gridded estimates derived using top-down and bottom-up geospatial methods.

**Results:**

In Zambia, vaccination estimates utilising the bottom-up approach to population estimation substantially reduced the number of districts with > 100% coverage of DTP3 and MCV1 compared to estimates using population projection and the top-down method. In Nigeria, results were mixed with bottom-up estimates having a higher number of districts > 100% and estimates using population projections performing better particularly in the South.

**Conclusions:**

Gridded demographic data utilising traditional and novel data sources obtained from remote sensing offer new potential in the absence of up to date census information in the estimation of RMNCAH indicators. However, the usefulness of gridded demographic data is dependent on several factors including the availability and detail of input data.

**Supplementary Information:**

The online version contains supplementary material available at 10.1186/s12913-021-06370-y.

## Background

The ‘leave no-one behind’ agenda focusing on equitable development is central to the Sustainable Development Goals (SDG) with universal health coverage (UHC) of quality services considered a key strategy to reach targets on reproductive, maternal, newborn child and adolescent health (RMNCAH). Substantial gains in the provision and use of health services have been seen in many low and middle income countries (LMICs), but progress has been uneven. Geographical location is often strongly associated with inequalities in health service utilisation where populations living in peripheral areas tend to have considerably lower utilisation and poorer health outcomes compared to more centrally located and urban populations [1–4]. Moreover, recent research suggests that there is a positive correlation between increasing coverage at national levels and increasing subnational inequalities in several sub-Saharan countries [5].

Representative household surveys such as Demographic Health Surveys (DHS) and Multiple Cluster Indicator Surveys are often used as data sources to measure the progress towards international and national RMNCAH targets including UHC. Key advantages of surveys are that they provide direct estimates of indicators and include measures of uncertainty. However, these surveys tend to be undertaken every 5 years and they are not necessarily synchronised with the reporting requirements of long term international targets and national health interventions plans. Furthermore, in countries with decentralised health systems, they are typically not representative at geographical administrative units relevant to planning and monitoring (e.g. districts).

LMICs have in recent years taken significant steps to meet the needs for data-driven planning and monitoring at subnational levels. Investments in district health management information systems (DHMIS) have resulted in increasing availability of timely data from health facilities providing regular information on the provision, service readiness and utilisation. Many RMNCAH indicators and related metrics used to plan and evaluate progress towards universal coverage, including utilisation such as antenatal care, delivery care and child vaccinations require integration of DHMIS data (numerators) with estimates of the relevant sub-population (denominators). These sub-populations typically include groups such as newborns, women of childbearing age (WoCBA) or children under 5 years old. In the absence of timely survey data at subnational levels, there has traditionally been a high reliance on population and housing censuses to produce estimates of population distributions by age and sex at subnational levels, since vital registration systems are often of poor quality or non-existent in many LMICs [6]. The advantage of using a census as a source for the calculation of denominators is that it includes the entire population, but since censuses are infrequent, information collected at the time of enumeration quickly becomes outdated [7]. Additionally, exact subnational boundaries for older census are often not well defined and can differ compared to digitised boundaries especially when different government ministries are responsible for different administrative unit levels. These can result in under- or over- coverage estimates (> 100%) of health and health care indicators at subnational levels as highlighted in previous research [7, 8].

Novel data and methodological advances are offering new opportunities to estimate population denominators at high spatial resolution that can be used in the monitoring coverage of RMNCAH interventions. Here we describe two different geospatial methods (top-down and bottom-up modelling) to estimate the numbers and distributions of population at high spatial resolution and highlight how gridded data can be used as a source of key catchment population counts at subnational levels. Using vaccination coverage in Zambia and Nigeria by districts, we show how these methods, as alternatives to population projections, can be combined with DHMIS data obtained from health facilities and highlight their potential in coverage estimation of RMNCAH interventions.

## Methods for measuring and mapping national population counts and key RMNCAH population subgroups at high spatial resolution

Population and housing censuses remain the core data source for the majority of countries globally in terms of obtaining small area demographics including population counts [9]. Moreover, they serve as the baseline from which projections are built for non-census years. Given the decadal implementation of national population censuses and the limitations of vital registry data sources, projections form the basis of the denominator population of many RMNCAH indicators [10, 11]. Population projections can be calculated in multiple ways. Simple projections from census baselines assuming a linear growth rate, or more complex methods, taking into account additional assumptions about age specific fertility and mortality, are generally undertaken by national statistical offices in LMICs to produce official estimates of age and sex structures in between census enumeration periods. However, the estimates are generally not undertaken at smaller geographical administrative units due to limited data on local patterns of fertility and mortality. Additionally, internal migration is rarely accounted for in such projections. This can have substantial impacts at subnational levels where rural to urban migration and/or movement across administrative boundaries are large. Furthermore, due to their size, complexity, costs or political and security constraints, many LMICs have struggled to conduct full censuses every decade, and the further such projections are undertaken away from the census baseline, the greater the uncertainty of the estimates [7, 12]. Finally, the importance of population estimates for resource allocation has resulted in political interference in enumeration, while in some cases poor implementation has led to unreliable numbers and populations being missed in some areas [13, 14]. This set of factors means that projections many years beyond a census baseline, in particular where there are issues with this baseline, can produce highly uncertain and inaccurate population estimates at subnational levels.

**Fig. 1:**
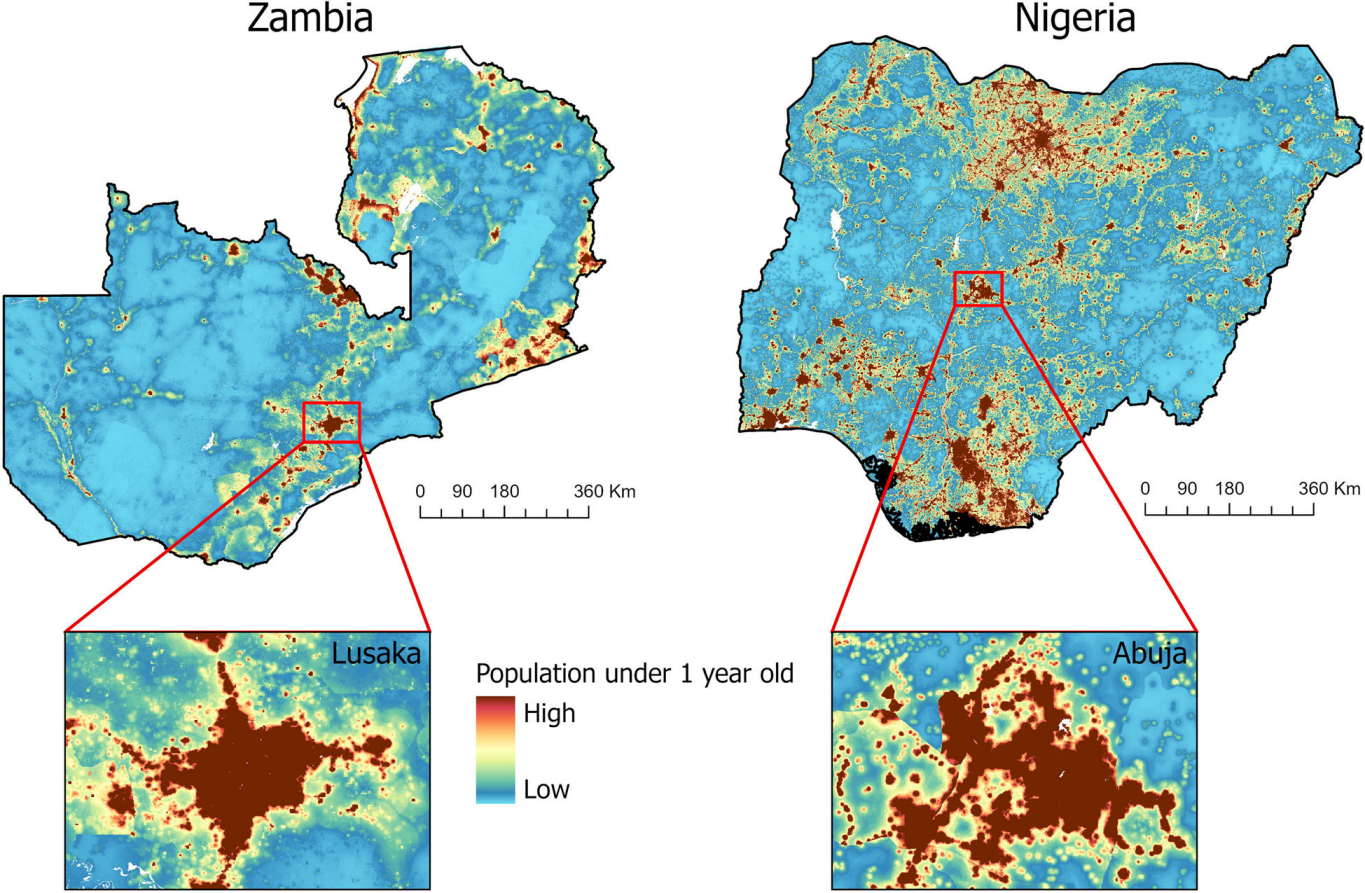
Estimated number of children aged 0–1 years old in Zambia and Nigeria in 2018 for each 100 × 100 metre grid cell, produced through application of a top-down modelling approach [23]

Geospatial methodologies using a combination of population data and satellite imagery have been put forward to compensate for some of these issues [7]. The past few decades have seen major advances in spatial detail and the availability of high resolution satellite imagery. Moreover, increasing computing power, along with advances in algorithm design using machine learning approaches have enabled automated processing of such imagery to extract high quality maps of building footprints, road networks, land use, neighbourhood types and other features related to population distributions across entire countries and continents [15–17]. To derive population estimates, increasingly sophisticated dasymetric methods have been developed for disaggregating census data (‘top-down’ models) to produce data at high spatial resolution [18, 19]. Such approaches have been applied for mapping population counts [20], age and sex structures [21], as well as births and pregnancies through the integration of subnational fertility data [22]. Figure 1 provides an illustration of gridded data produced through top-down modelling, showing the estimated number of children under 1 year in Zambia and Nigeria in 2018.

To counteract the tendency for poor population projections from long past censuses, a growing area of research is now focussing on the development of census-independent methods for population estimation that are built on detailed geospatial datasets and statistical models (‘bottom-up’ models). Through the use of small area sample enumerations and the exploration of the relationships between population densities measured in these enumeration data and geospatial covariate data, statistical models such as Bayesian hierarchical approaches fitted using integrated nested Laplace approximation or other methods are built to predict population numbers in unsampled areas, together with uncertainty intervals, based on covariate and spatial relationships [7, 24]. The approaches can also be adapted to situations where an incomplete census has been undertaken and estimates are required for areas where enumeration was not possible. Such approaches have been developed and applied to produce cross-sectional estimates in Nigeria, Zambia, Democratic Republic of the Congo, Colombia and Afghanistan [25–30]. Cross-validation efforts have shown the accuracy and value of such approaches, and validation with independent datasets is being planned in multiple settings.

## Gridded demographic mapping and its applications

Aside from the simple projections from census data at national or province levels that still form the basis of most official population data in between census periods, each of the geospatial approaches outlined above can produce population estimates at grid squares of e.g. 1x1km or 100x100m.

Gridded data have a range of advantages over the production of estimates at administrative geographical unit levels. Firstly, by producing estimates for each grid square across a country, the spatial variations in population densities and characteristics that exist across a landscape can be better captured. In the SDG ‘leave no-one behind’ era and in the context of UHC, this has significant advantages in terms of identifying and mapping populations that can often be missed in large area totals or averages, ensuring that everyone is counted and policies can be tailored accordingly. Secondly, they facilitate the ease of integration with DHMIS and infrastructure datasets that are key to RMNCAH interventions at different spatial scales including according to health facility catchment area. Health facilities are increasingly being mapped using the global position system across LMICs [31, 32], and it is challenging to measure the population served or within reach of these facilities using population estimates mapped to administrative provinces or districts. Gridded demographic data present a more suitable format for the flexible provision of, for instance, estimates of numbers of WoCBA or total number of births residing within a certain distance or travel time of a facility providing comprehensive emergency obstetric and neonatal care [33]. Finally, the flexibility of gridded data at high spatial resolution means that it can be summarised to construct population estimates at district, province or any operational unit required using geographic information system software. While studies using gridded demographic data on population and births to calculate RMNCAH service utilisation are limited, they have more frequently been used in the estimation of availability of and travel time to health facilities including emergency obstetric and newborn care [34–36].

While gridded population data do have some significant advantages over data at larger administrative units, it is important to keep in mind the uncertainties inherent in data at such fine spatial scales [18]. Uncertainty intervals in estimates tend to be much larger at small scales and reduce as the data are aggregated. Recent work applying Bayesian statistical models to capture uncertainty provides quantitative measures of uncertainty in population estimates at grid square, district, province and national levels, highlighting how uncertainty intervals shrink as data is aggregated, and the need to account for this in applications [7, 24, 37].

## Using gridded population data to produce vaccination coverage estimates in Zambia and Nigeria

In the absence of representative household survey data on RMNCAH coverage indicators at subnational levels, LMICs are exploring the use of existing data where the numerator is derived from a different data source (typically DHMIS) compared to the denominator (typically a census). Using this approach, it is not uncommon to find subnational estimates of health intervention coverages reported as being > 100% [38] and sources of error can be associated with both the numerator and the denominator [8]. Here, we provide coverage estimates for diphtheria, pertussis and tetanus vaccine dose 3 (DTP3) and measles vaccine dose (MCV1) in Nigeria (774 districts) and Zambia (110 districts) to exemplify how gridded population data can be used as alternatives to population estimates obtained from nationally produced projections. Nigeria and Zambia were selected because district level information on vaccinations and data using all three methods for estimating the denominators (national projections from censuses, top-down modelling and bottom-up modelling) were available for analysis. For the numerators, we obtained district level data on vaccine doses delivered for DTP3 and MCV1 for 2018 sourced from each country’s respective DHMISs, as reported to the World Health Organisation (WHO) [38]. The numerators are held fixed across each respective vaccination coverage estimate for each country to show how the three different denominator estimates of the population vary depending on the method applied. The methods used to estimate the denominator population used data from 2016/2017, 2018 or 2019 depending on data availability. The denominator estimates derived using official census-based population projections were reported by each country to the WHO for the year 2018 [38]. The top-down geospatial population estimates for 2018 for both countries produced by WorldPop were obtained through dasymetric methods using subnational projections from census data [23], and the bottom-up population estimates were derived from geospatial modelling using a Bayesian approach implemented for the Geo-Referenced Infrastructure and Demographic Data for Development (GRID3) programme [26, 39, 40]. The bottom-up estimates for Zambia used data from 2019 and the bottom- up estimates for Nigeria used data from 2016/17. Because estimates of 12–23 months old children commonly used to produce DTP3 and MCV1 indicators are not available, estimates of 0–1 years old are used as proxies. For top-down and bottom-up modelled datasets in both countries, the 0–1 year old proportions are defined using subnational data from household surveys [21]. The bottom-up population estimates are the only modelled estimates that provide uncertainty metrics for the population denominator. Uncertainty estimates of DTP3 and MCV1 coverage are measured as the difference between the lower and upper ends of the 95% credible intervals (Supplementary Fig. 1). Because true vaccination coverage in Zambia and Nigeria is unknown at the district level, the performance of the population estimates in regards to under-coverage cannot be assessed, but the frequency of over-coverage defined as districts with > 100% coverage can be analysed and compared across the estimates using the different denominator source. While > 100% coverage does not necessarily mean there is an error in the denominator, it does indicate a high likelihood of problems, for example with more children receiving doses than estimated to be living in the district, suggesting inaccuracies in the numerator.

**Fig. 2:**
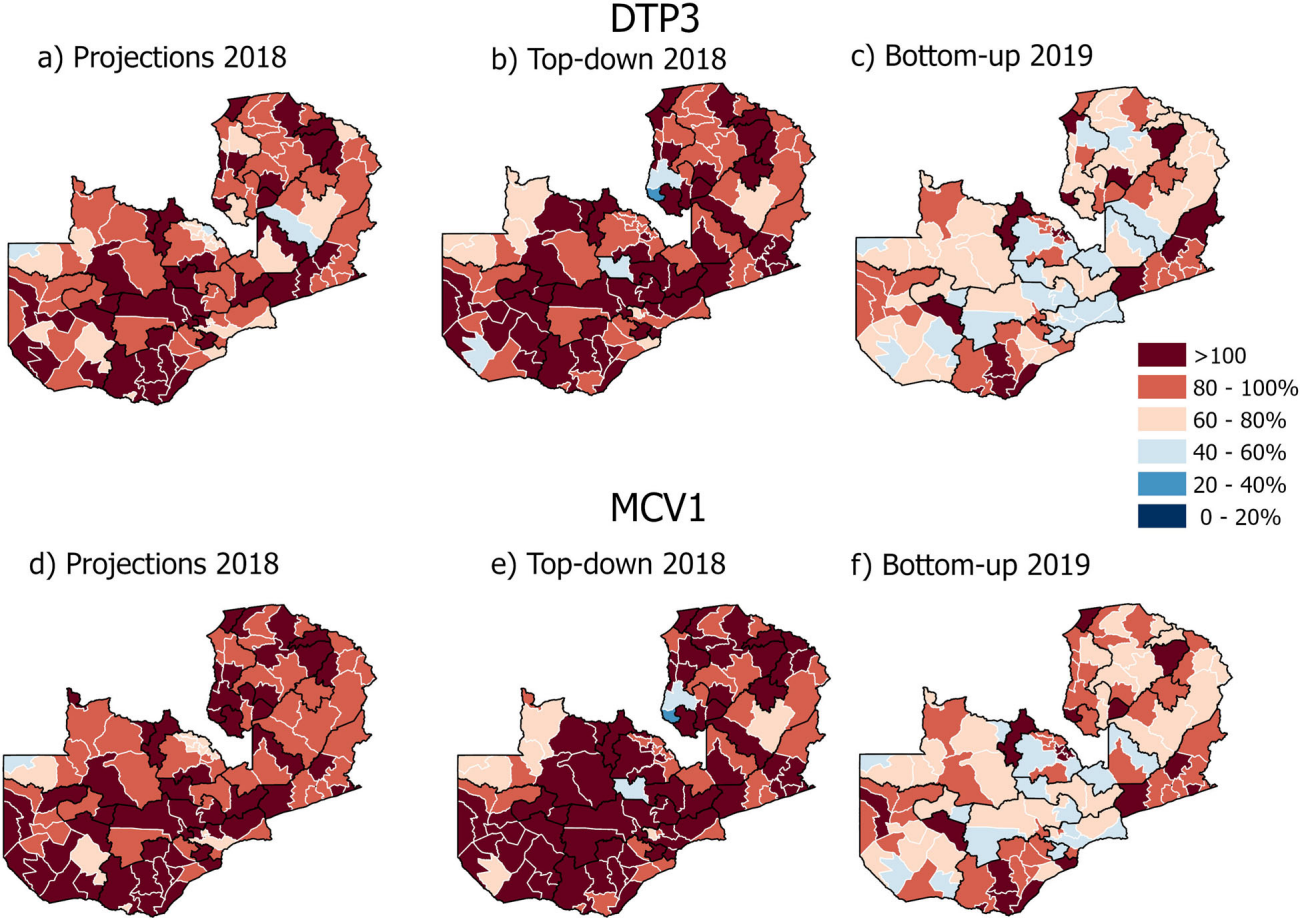
Estimated district coverage maps of diphtheria, pertussis and tetanus vaccine third dose (DTP3) and measles first dose (MCV1) for Zambia. Numerator: DTP3 and MCV1 vaccination doses from DHMISs, as reported to the WHO (maps **a**, **b**, **c**, **d**, **e** and **f**) [38]. Denominators: official population projections from the last census as reported to WHO (map **a** and **d**) [38]; WorldPop modelled top-down population estimate (map **b** and **e**) [23]; and GRID3 modelled bottom-up population estimate (map **c** and **f**) [26, 39]

**Fig. 3:**
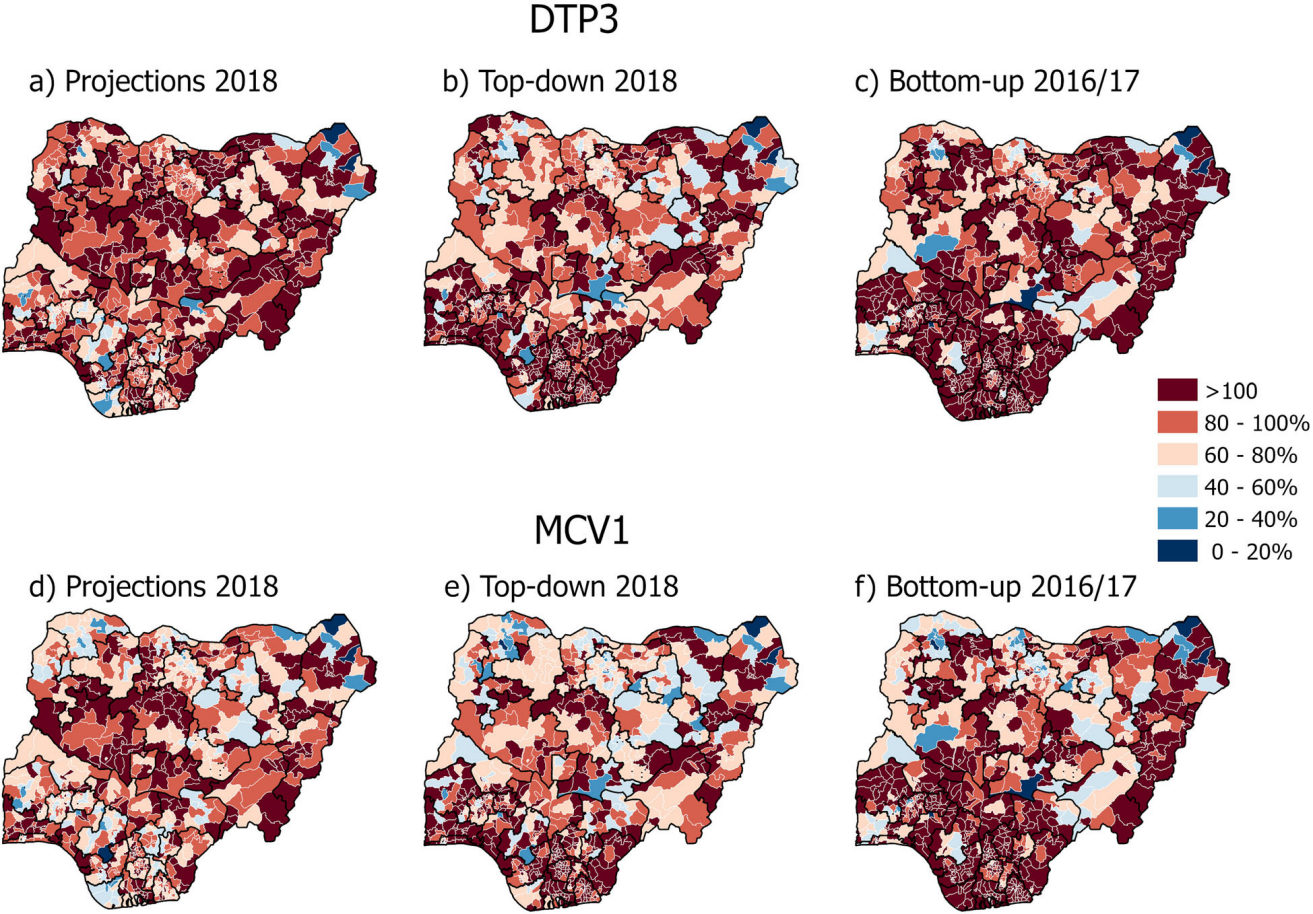
Estimated district coverage maps of diphtheria, pertussis and tetanus vaccine third dose (DTP3) and measles first dose (MCV1) for Nigeria. Numerator: DTP3 and MCV1 vaccination doses from DHMISs, as reported to the WHO (maps **a**, **b**, **c**, **d**, **e** and **f**) [38]. Denominators: official population projection from the last census as reported to WHO (map **a** and **d**) [38]; WorldPop modelled top-down population estimate (map** b** and **e**) [23]; and GRID3 modelled bottom-up population estimate (map **c** and **f**) [36, 40]

Figures 2 and 3 show coverage estimates of DTP3 and MCV1 for Zambia and Nigeria respectively using population projection (maps a and d), top-down model (map b and e) and bottom-up model (map c and f). In both countries, the vaccination estimates vary considerably according to the denominator estimates used, but patterns within countries also vary. Figure 2 shows that in Zambia, vaccination estimates utilising the bottom-up approach to population estimation substantially reduce the number of districts with > 100% coverage compared to estimates using population projection and the top-down method (maps a-f). For DTP3, the bottom-up approach yields 13 districts, the population projection yields 37 districts and the top-down yields 49 districts with vaccination coverage > 100%. For MCV1, the corresponding figures are 18, 51, and 61 districts with > 100% vaccination coverage for bottom-up, projection and top-down estimates respectively (Supplementary Table 1).

Figure 3 (maps a-f) shows that DTP3 and MCV1 coverage estimates in Nigeria vary considerably according to different methods of population estimation, with similar patterns evident for both vaccination programmes. With the exception of some districts, the bottom-up estimate has substantially more districts with > 100% vaccination coverage compared to the projection and top-down estimates. For example, the bottom-up estimate yields 509 districts with > 100% coverage compared to 282 districts using projection and 405 districts using the top-down estimate (see Supplementary Table 2 for these and corresponding estimates for MCV1). Furthermore, a geographical pattern suggesting a North- South divide in vaccination coverage of > 100% according to the different population estimation methods (top-down and projection) can be identified. Figure 3 (maps a, b, d and e) and Supplementary Table 2 show that in the North west, North central and the North east, the population projection yields 46 more districts with > 100% DTP3 coverage compared to top-down estimation (*n* = 178 districts and 132 districts respectively). Conversely, the top-down method of population estimation produces more districts with vaccination coverage estimates of > 100% in the South, South west and South east of the country compared to those derived using projections. For DTP3, the top-down method has 163 more districts with coverage estimates with > 100% compared to population projections (*n* = 273 and 104 districts respectively). Similar patterns are observed for MCV1 vaccination coverage estimates (Supplementary Table 2).

## Conclusions

While household surveys are likely to continue to play a prominent role to measure the progress towards international and national RMNCAH targets including UHC in the future, the potentials of other sources should be explored to produce more frequent estimates at geographical administrative levels where survey data are not representative. Here we have highlighted how traditional demographic data from censuses can be complemented by new technologies to improve the spatial detail and recency of population estimates. Moreover, they can support the production of gridded outputs to facilitate ease of integration with DHMIS data when estimates obtained through population projections are considered too unreliable to be used in health coverage estimation.

RMNCAH coverage indicators require accurate population data to be estimated at subnational levels. Figures 2 and 3 (maps a and d) highlighted the challenges that governments face in producing subnational vaccination coverage estimates, when data on vaccination doses are combined with simple projections from census data collected more than a decade previously, with many districts showing coverage estimates of over 100%. This could be due to, for example, reporting errors inherent in numerators or associated with coverage errors in the denominator, including inaccurate assumptions that affect population growth and change [8]. It can also be due to the health care seeking behaviour of the population living in one subnational administrative catchment area crossing its border to obtain health services in another area [41], particularly in cases where administrative units with > 100% coverage have neighbouring units with large underestimates. It should also be noted that coverage estimates of < 100% can also be inaccurate, though more difficult to determine, since they are within the feasible range for coverage estimation. We did not distinguish coverage estimates that exceeded 100% by different amounts (for example, 101% was treated the same as 200%).

Keeping the numerator fixed, results contained in Figs. 2 and 3 on vaccination coverage exemplify the advantages and limitations of the applicability of geospatial population modelling in the monitoring of RMNCAH interventions. The switch from official population projections to model based estimates produced using top-down (map b and e) and bottom-up (map c and f) methods in Nigeria results in different vaccination coverage patterns, with fewer districts showing coverage estimates over 100% in the north and centre, but interestingly, more districts breaching this threshold in the south. The reasons for these variations are complex, with many factors driving differences in estimates. Firstly, questions over the Nigeria 2006 census that forms the baseline of the WHO and top-down modelling have been raised regularly [42]. Secondly, the approaches for projecting these up until recent years and breaking them down by age group differ between the two geospatial approaches, with the dataset making use of subnationally varying growth rates and age structure data from recent household surveys [21]. Thirdly, the settlement map (LandScanHD) used in the bottom-up approach in Nigeria was mostly based on 2014 satellite imagery and therefore omits newly built up areas [30], and areas mapped as non-residential were excluded (e.g. mapped as zero population) because no population data were available from those areas [25]. Fourthly, the different years represented by the available denominator datasets likely had an impact, given high and varying age specific fertility rates across Nigeria. Finally, uncertainties in the numerator data exist, with the precise district boundaries used in collating vaccination delivery numbers being unclear, meaning that those used to both map out the WHO coverage estimates and summarise the modelled estimates could have been different, contributing to errors in mapping coverages. All of these factors contribute to uncertainties in estimates of both the denominators and numerators, emphasising the need to capture and communicate these where possible, as well as the need for further research into each source of uncertainty.

While top-down population estimates suffer from the same limitations and uncertainties as census projections, this could be partially compensated for by the method’s utilisation of recent geospatial datasets on settlements, buildings and infrastructure. Bottom-up models also incorporate such information, however it is important to note that the precision of the population predictions in such models is largely dependent on the numbers, locations, sizes and representivity of the sample area enumerations in addition to recency of the high resolution satellite imagery used [7, 30].

The bottom-up estimates used here utilise a Bayesian modelling framework to undertake the measurement of uncertainty [24]. Efforts have been made recently to estimate and map similar uncertainty ranges in vaccination coverages from household surveys [43, 44], and future work should be directed towards trying to capture such uncertainties from DHMIS data. Reducing uncertainties and improving predictions in model-based estimates require a coordinated effort to incorporate the latest population enumerations from household surveys, high resolution settlement maps from recent satellite imagery, and other geospatial data into population models.

In Zambia, the top-down estimates yielded a larger number of districts with > 100% vaccination coverage compared to the estimates using population projections. This could be due the simple subnational projections used to produce the input count data [45]. Conversely, the bottom-up estimates greatly reduced the number of districts at > 100% compared to the population projections (and the top-down estimates). Compared to the bottom-up estimates for Nigeria, the estimates for Zambia were based on more recent building footprints, mostly from 2018 and 2019. Furthermore, compared to Nigeria, multiple data sources were obtained to achieve good national coverage of sample locations as well as representation of non-residential settlement types. While the overall reduced number of districts with > 100% vaccination coverages in Zambia suggests that the bottom-up estimates would be the favourable denominator, there may also be districts with underestimates of vaccination coverage due to overestimated district level population counts. For both Zambia and Nigeria, underestimation or overestimation due to the denominator is most likely in areas where there is high uncertainty in the bottom-up estimates (Supplementary Fig. 1).

There have been limited attempts to systematically evaluate the predictions of the population estimates using any of the methodologies discussed here. Validation of both top-down and bottom-up modelled population estimates have largely involved cross-validation so far [46, 47], and there is a need for additional assessments using independent datasets. Part of the challenge is to identify accurate population data sources to compare the outputs of the different methodologies with up-to-date population enumerations immediately utilised to update and improve models. A thorough approach to validation will require the comparison of outputs in several countries, following previous cross-validation efforts, as different methods may perform better or worse depending on country specifics. Such an assessment of modelling method accuracies would facilitate narrowing down the drivers behind some of the differences seen in Figs. 2 and 3.

The approaches to the production and use of modelled population datasets outlined above represent an alternative for the production of key RMNCAH metrics for small areas where existing demographic data are outdated, unreliable, incomplete or non-existent. Their potentials are not limited to district level estimates as demonstrated here. The methodology can also be applied to other small areas, for example, in under-researched urban settings such as slum areas where low service use and poor health outcomes are masked by high urban area averages. The results for Nigeria show that they only represent one component of the challenge in producing robust and reliable metrics with much work still to be done in understanding sources of uncertainty and inaccuracy. Further still, the integration of such new datasets and approaches into routine use in health systems will require significant capacity strengthening efforts in some countries where expertise in geographical information systems and spatial data are limited. Finally, population data can be highly sensitive and political, and resistance to the use of alternative estimates that vary substantially from official estimates can be common, placing a further barrier to the adoption and widespread use of such data. Nevertheless, the advantages of such gridded population datasets outlined above are leading to their increasing use, with local universities and multiple international initiatives supporting ministries of health, national statistics offices and others across LMICs to explore the use of these new forms of data in building health information systems.

## Supplementary Information

**Additional file 1: Figure S1.** Uncertainty of DTP3 and MCV1 in Nigeria in 2016/7 (map a and b) and Zambia 2019 (map c and d) using a bottom-up approach to population estimation for children aged 0–1 years old. Uncertainty is displayed as the difference between the lower and upper 95% credible boundaries of the vaccination coverage estimates. Numerator: DTP3 and MCV1 vaccination doses from each country’s DHMISs, as reported to the WHO (maps a, b, c and d) [38]. Denominator: GRID3 modelled bottom-up population estimate for Nigeria (map and b) [39, 40] and GRID3 modelled bottom-up population estimate for Zambia (map c and d) [26, 39].

**Additional file 2: Table S1.** Number of districts in Zambia where vaccination coverage for DTP3 and MVC1 is > 100%. Numerator: DTP3 and MCV1 vaccination doses from each country’s DHMISs, as reported to the WHO [38]. Denominator: official population projections from the last census as reported to WHO [38]; WorldPop modelled top-down population estimates [23]; and GRID3 modelled bottom-up population estimate [26, 39].

**Additional file 3: Table S2.** Number of districts by region in Nigeria where vaccination coverage for DTP3 and MVC1 is > 100%. Numerator: DTP3 and MCV1 vaccination doses from DHMISs, as reported to the WHO [38]. Denominators: official population projection from the last census as reported to WHO [38]; WorldPop modelled top-down population estimate [23]; and GRID3 modelled bottom-up population estimate [39, 40].

## Data Availability

The data on DTP3 and MCV1 for Nigeria and Zambia that support the findings of this study are available from the WHO, but restrictions apply to the availability of these data and so are not publicly available. Data are however available from the authors upon reasonable request and with permission of the WHO and relevant government authorities in Nigeria and Zambia. The data on the population projections for Nigeria and Zambia that support the findings of this study are available from the WHO, but restrictions apply to the availability of these data and so are not publicly available. Data are however available from the authors upon reasonable request and with permission of the WHO and relevant government authorities in Nigeria and Zambia. The datasets using a top-down method (WorldPop) to estimate the population aged 0–1 years old in Nigeria and Zambia used for the analysis of this study are available here: WorldPop School of Geography and Environmental Science, University of Southampton, Department of Geography and Geosciences, University of Louisville, Departement de Geographie, Universite de Namur and Center for International Earth Science Information Network (CIESIN), Columbia University. The spatial distribution of population in 2018, Nigeria. Global High Resolution Population Denominators Project; 2018. Funded by The Bill and Melinda Gates Foundation (OPP1134076). 10.5258/SOTON/WP00646 The datasets using a bottom-up method (GRID3) to estimate the population aged 0–1 years old in Nigeria used for the analysis of this study are available here: WorldPop School of Geography and Environmental Science, University of Southampton. Bottom-up gridded population estimates for individual age-sex groups in Nigeria, version 1.2.1. 2020. 10.5258/SOTON/WP00661 The datasets using a bottom-up method (GRID3) to estimate the population aged 0–1 years old in Zambia used for the analysis of this study are available here: WorldPop. Bottom-up gridded population estimates for Zambia, version 1.0. WorldPop, University of Southampton. 2020; 10.5258/SOTON/WP00662

## Abbreviations

DHMIS: District Health Information System
DHS: Demographic Health Survey
DTP3: Diphtheria, Pertussis and Tetanus Vaccine Dose 3
GRID3: Geo-Referenced Infrastructure and Demographic Data for Development
LMIC: Low and Middle Income Countries
MCV1: Measles Vaccine Dose 1
RMNCAH: Reproductive, Maternal, Newborn, Child and Adolescent Health
SDG: Sustainable Development Goal
UHC: Universal Health Coverage
WHO: World Health Organisation
WoCBA: Women of Child Bearing Age
