# Supplementary figures and images for: A review of geospatial methods for population estimation and their use in constructing reproductive, maternal, newborn, child and adolescent health service indicators
Source: BMC Health Serv Res. 2021 Sep 13;21(Suppl 1):370. doi: 10.1186/s12913-021-06370-y (PMC8436450; doi:10.1186/s12913-021-06370-y)

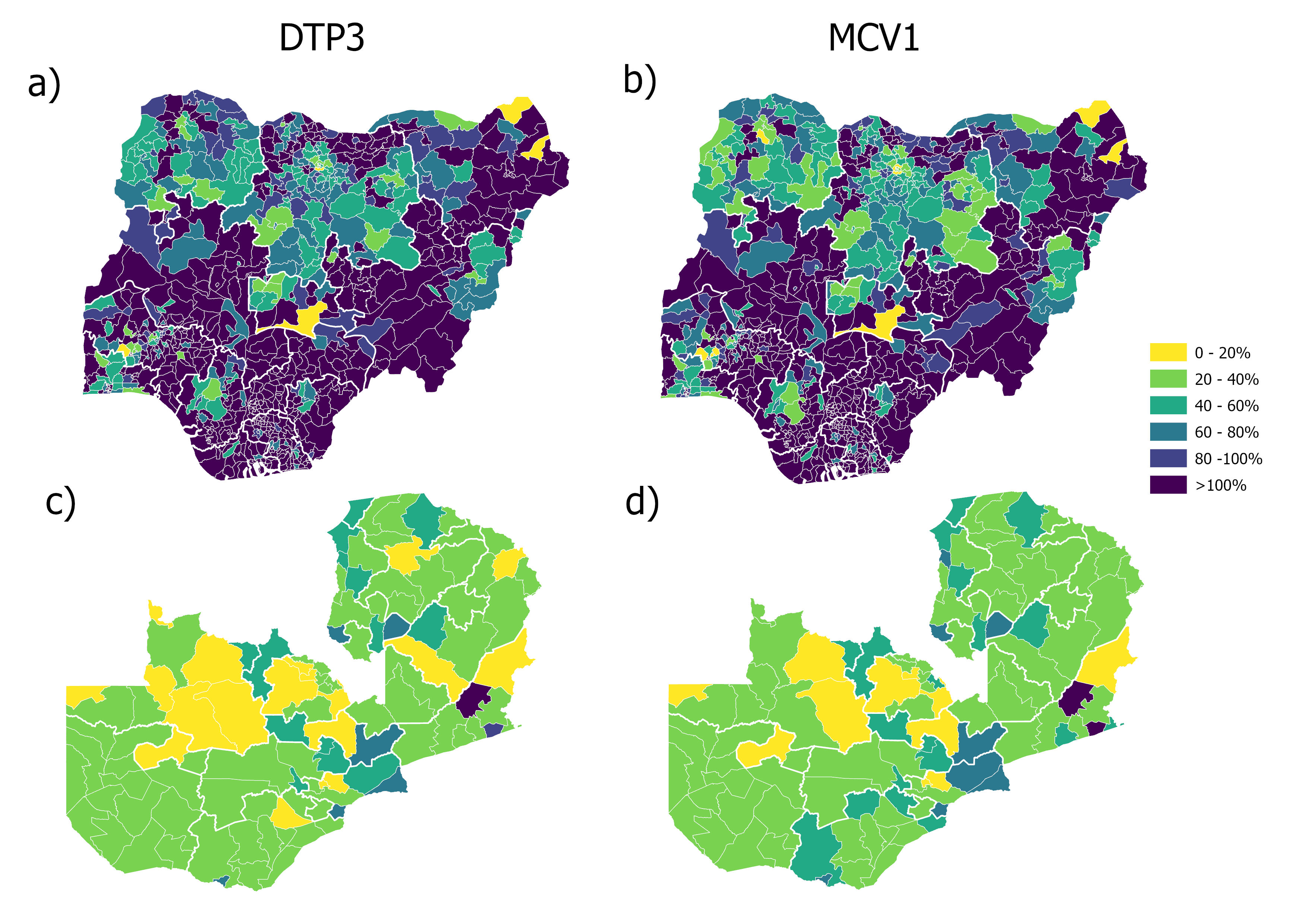

Supplement: Supplementary file 1 — Additional file 1: Figure S1. Uncertainty of DTP3 and MCV1 in Nigeria in 2016/7 (map a and b) and Zambia 2019 (map c and d) using a bottom-up approach to population estimation for children aged 0–1 years old. Uncertainty is displayed as the difference between the lower and upper 95% credible boundaries of the vaccination coverage estimates. Numerator: DTP3 and MCV1 vaccination doses from each country’s DHMISs, as reported to the WHO (maps a, b, c and d) [38]. Denominator: GRID3 modelled bottom-up population estimate for Nigeria (map and b) [39, 40] and GRID3 modelled bottom-up population estimate for Zambia (map c and d) [26, 39]. [file 12913_2021_6370_MOESM1_ESM.jpg]
